# Does disease start in the mouth, the gut or both?

**DOI:** 10.7554/eLife.45931

**Published:** 2019-03-19

**Authors:** Andrei Prodan, Evgeni Levin, Max Nieuwdorp

**Affiliations:** 1 Department of Vascular MedicineAmsterdam University Medical CenterAmsterdamThe Netherlands

**Keywords:** microbiome, gastrointestinal tract, colorectal cancer, rheumatoid arthritis, metagenomics, Human, Other

## Abstract

Oral bacteria colonize the gut more frequently than previously thought.

**Related research article** Schmidt TSB, Hayward MR, Coelho LP, Li SS, Costea PI, Voigt AY, Wirbel J, Maistrenko OM, Alves RJ, Bergsten E, de Beaufort C, Sobhani I, Heintz-Buschart A, Sunagawa S, Zeller G, Wilmes P, Bork P. 2019. Extensive transmission of microbes along the gastrointestinal tract. *eLife* **8**:e42693. doi: 10.7554/eLife.42693

Different sites inside the body and on its surface are home to distinct ecosystems of bacteria and other microbes (Sender et al., 2016). The microbes in these ecosystems, which are known as microbiomes, are more than just microscopic hitchhikers; they constantly interact with their human host. The gut microbiome is one of the largest microbiomes and it is generally regarded as a friend: it helps train the immune system, keeps dangerous colonizers away, and produces small molecules that nurture the cells that line the colon. However, the microbiome can turn into a foe if the host–microbe relationship is thrown off balance. Such an imbalance can cause gut microbes to produce carcinogenic toxins, or trigger gut inflammation and metabolic problems. Thus, an in-depth understanding of the factors that shape the human microbiome is key to preventing and controlling disease.

In the past it was thought that the microbes usually found in the mouth only transfer to the gut in people with specific diseases, such as rheumatoid arthritis, inflammatory bowel disease and colorectal cancer (Valdes et al., 2018). In healthy individuals, on the other hand, it was thought that stomach and bile acids reduce the number of viable oral bacteria that reach the gut to an extent that prevents any significant colonization (Martinsen et al., 2005; Ridlon et al., 2014).

Now, in eLife, Peer Bork and colleagues – including Thomas Schmidt and Matthew Hayward of the European Molecular Biology Laboratory (EMBL) as joint first authors – report compelling evidence that bacteria are transmitted from the oral environment to the gut far more often than was previously thought (Schmidt et al., 2019). They show that while the oral and gut microbiomes of an individual have distinct, unrelated compositions, strains of oral bacteria colonize the gut far more often than explained by chance alone.

Schmidt et al. – who are based at EMBL and other institutes in Germany, France, Luxembourg, Denmark, Switzerland, China and the US – used DNA shotgun sequencing data obtained from the saliva and stools of 470 people to track oral-fecal strain transmission. Building profiles of variations in single nucleotides in the DNA sequences allows the microbial strains in the samples to be identified (Li et al., 2016). From these profiles, Schmidt et al. built mathematical models that indicate the likelihood of a strain transferring from mouth to gut within a particular individual. They found that 74 of the 125 microbial species frequently found in both the mouth and the gut showed evidence of oral-gut transmission in all individuals: these species included a number of bacteria that are highly prevalent in the mouth, such as *Streptococcus*, *Veillonella*, *Actinomyces* and *Haemophilus*. Moreover, about 22 bacterial species appeared to be transmitted from mouth to gut in at least some individuals, including strains from all species of *Prevotella*, one of the major fecal bacteria.

However, there are some caveats to the results that merit caution. It is difficult to differentiate between bacteria that truly reside in – and actively colonize – the gut, and those that move passively through it as either live cells or dead cell DNA. The oral cavity contains many orders of magnitude fewer bacteria than the gut. In addition, the low pH of stomach acid usually reduces the viability of oral bacteria significantly, and enzymes in the small intestine degrade at least some of the released bacterial DNA (Liu et al., 2015). One way to assess the viability of bacteria is by looking at the ‘peak-to-trough ratio’ in DNA sequencing reads (Gao and Li, 2018; Korem et al., 2015), which estimates the proportion of bacteria with active DNA replication forks. These bacteria are actively multiplying, suggesting that they have colonized their environment. However, Schmidt et al. had insufficient sequencing depth in their data to reliably apply this method.

Schmidt et al. argue that the most plausible interpretation for their results is that oral strains successfully colonize the gut, but gut strains do not generally colonize the oral cavity. In line with this, several oral bacteria that are known to be relevant to human disease, including *Haemophilus*, *Aggregatibacter* and *Streptococci viridans* (which are associated with endocarditis), were found to be highly transmissible.

To study the link between disease and oral-gut transmission further, Schmidt et al. examined samples from colorectal cancer patients. Compared with healthy individuals, patients diagnosed with cancer had higher transmission rates of bacteria from the oral cavity to the gut, especially for strains associated with colorectal cancer, such as *Fusobacterium nucleatum*, *Parvimonas micra* and *Peptostreptococcus stomatis. F. nucleatum* underwent particularly frequent transmission and could be used to predict overall high transmission rates in these individuals. The results thus support a link between the oral and gut microbiome in the context of colorectal cancer (Flemer et al., 2018; Flynn et al., 2016).

By challenging the established view of the relationship between the oral microbiome and the gut microbiome in humans, the work of Schmidt et al. is a step towards a better understanding of the links between the bacterial strains found in the mouth and intestinal tract and their relationship with human disease.
